# A Multifunctional Mesoporous Janus Separator With Optimized Mass Transport Regulation Enabling 800‐Cycle Lithium‐Oxygen Batteries

**DOI:** 10.1002/advs.77588

**Published:** 2026-09-13

**Authors:** Hailiang Mu, Anlong Liu, Kun Luo, Yaming Pang, Chengde Xie, Xiaoteng Liu, Zhihong Luo, Guangbin Zhu, Yucheng Wang, Qirong Liu, Yongbing Tang

**Affiliations:** ^1^ Jiangsu Province Engineering Research Centre of Intelligent Manufacturing Technology for the New Energy Vehicle Power Battery School of Materials Science &Engineering Changzhou University Changzhou People's Republic of China; ^2^ Advanced Energy Storage Technology Research Center Shenzhen Institutes of Advanced Technology Chinese Academy of Sciences Shenzhen People's Republic of China; ^3^ School of Mechanical Engineering Shanghai Jiao Tong University Shanghai People's Republic of China; ^4^ Geely Automobile Research Institute (Ningbo) Co., Ltd. Ningbo People's Republic of China; ^5^ School of Engineering, Physics and Mathematics, Faculty of Science and Environment Northumbria University Newcastle upon Tyne UK; ^6^ Guangxi Key Laboratory of Optical and Electronic Materials and Devices Guilin University of Technology Guilin People's Republic of China; ^7^ School of Environmental Science and Engineering Tianjin University Tianjin People's Republic of China

**Keywords:** lithium‐oxygen battery, mass transport regulation, mesoporous Janus separator, shuttle effect, ultrahigh cycling stability

## Abstract

Lithium‐oxygen batteries (LOBs) deliver ultrahigh theoretical specific energy but suffer from limited cycle life caused by lithium dendrite growth, anode corrosion, and cathode passivation. Developing interfacially compatible functional separators is an effective solution, yet coordinating fast ion conduction and multi‐species mass transport regulation remains challenging. We design an asymmetric multifunctional mesoporous Janus separator (mGP), consisting of a ∼ 3.16 nm mesoporous silica (mSiO_2_) layer and dense, hydrophobic, mechanically tough polyurethane (PU). Via steric confinement, the mSiO_2_ nanochannels optimize mass transport behavior: they homogenize Li^+^ flux and accelerate ion transport, suppress intermediate crossover to alleviate the shuttle effect and cathode passivation. The PU layer prevents dendrite piercing, buffers lithium volume variation, and suppresses water‐ and intermediate‐triggered anode corrosion via hydrophobicity. Li||Li symmetric cells with mGP run stably for 1300 h at 0.1 mA cm^−2^. LOBs based on mGP achieve exceptional cycling stability: over 800 cycles at 1000 mAh g^−1^ and 100 cycles at 3000 mAh g^−1^, outperforming traditional glass fiber (GF) separators. This work provides a feasible strategy for constructing high‐performance long‐cycle LOBs through deliberate mass transport optimization.

## Graphical abstract

To resolve the fundamental cycling decay of lithium‐oxygen batteries arising from lithium dendrite propagation, anode corrosion, and cathode passivation, we fabricate a mesoporous silica/polyurethane Janus separator integrating interfacial protection and mass transport modulation, which markedly enhances battery cycling stability and cycle life.

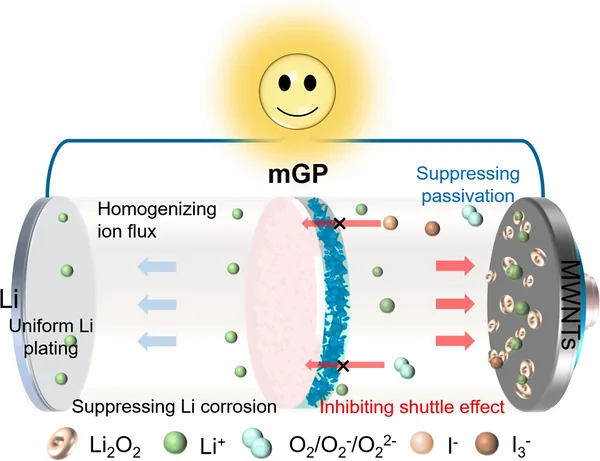

## 1 Introduction

LOBs have emerged as a highly promising energy storage technology for diverse applications, including electric vehicles and spacecraft, owing to their high theoretical specific energy (∼ 3600 Wh kg^−1^) [1, 2, 3]. However, their practical development is currently hindered by a formidable challenge of poor cycling performance, primarily stemming from dendrite growth and surface corrosion of Li anodes, as well as cathode passivation [4, 5, 6, 7]. For example, non‐uniform ionic flux through separators is prone to accelerate Li dendrite growth and subsequent volume deformation of Li anodes, thereby compromising the cycling stability of LOBs [8, 9]. Furthermore, intermediate products such as superoxide ions (O_2_^−^), peroxide ions (O_2_^2−^), singlet oxygen (^1^O_2_), along with trace amounts of water (H_2_O) and dissolved oxygen (O_2_) in the electrolyte, can corrode Li anodes, thus increasing the interfacial resistance of LOBs [10, 11]. Numerous design strategies have been proposed to improve the cycling stability of LOBs, including electrolyte chemistry, interfacial modification, and electrode engineering. For instance, approaches such as developing solid electrolytes, artificial protective coatings, and alloying anodes aim to facilitate the uniform Li^+^ stripping/plating [12, 13, 14]. In the realm of electrolyte chemistry, the use of redox mediators, including lithium iodide (LiI), 1,3‐dimethylimidazolium iodide, and lithium bromide, can accelerate the decomposition of discharge products [15, 16, 17, 18, 19]. Notably, although existing strategies have demonstrated some efficacy in mitigating dendrite growth, improving the electrochemical reversibility of discharge products, and reducing cathode passivation, LOBs still encounter substantial challenges in achieving high cycling stability exceeding 500 cycles at a discharge capacity of ≥ 1000 mAh g^−1^ [15, 16, 17, 18, 19, 20, 21, 22, 23, 24, 25, 26, 27].

Alternatively, significant efforts have been devoted to the elaborate design of separators with excellent hydrophobicity and gas impermeability, aiming to suppress the shuttle effect of intermediate products between both electrodes and enhance the cycling stability of LOBs [28]. However, such separators typically exhibit relatively low ionic conductivity and have emerged as a critical bottleneck limiting the improvement of reaction kinetics in LOBs [28, 29]. Hence, it remains a formidable challenge for separators to simultaneously realize rational multi‐species mass transport regulation and fast ionic conduction. Recently, mSiO_2_ has garnered considerable attention due to its tunable morphology, particle size, and pore size [30, 31, 32, 33, 34, 35, 36]. Meanwhile, mSiO_2_ possesses a high specific surface area and pore volume. The tunable pore structure and abundant pore volume are expected to endow mSiO_2_ with excellent size sieving‐mediated mass transport capability, thereby facilitating the transport of target Li^+^ in LOBs. Additionally, the surface interaction between mSiO_2_ and Li^+^ is anticipated to enhance the desolvation capability of Li^+^ and increase their transference number. Therefore, mSiO_2_ represents a promising material for addressing the aforementioned challenges, demonstrating significant application potential in LOBs.

Herein, a novel multifunctional mesoporous Janus separator (mGP) featuring an asymmetric structure is rationally engineered for high‐performance LOBs. This separator is primarily composed of two layers: one is a mSiO_2_ layer with a specific pore structure and a pore size of approximately 3.16 nm, and the other is a dense, hydrophobic PU layer with high mechanical strength. mSiO_2_ nanoparticles are densely immobilized on the GF separator substrate, forming a robust framework matrix. The nanochannels within the mSiO_2_ layer achieve differential mass transport via the size‐sieving effect and efficiently accelerate the rapid transmembrane transport of Li^+^. Meanwhile, the mSiO_2_ matrix binds reactive intermediates including O_2_^−^, effectively curbing the shuttle effect and cathode passivation. Furthermore, these nanochannels homogenize ionic flux distribution and accordingly elevate the electrochemical reversibility of LOBs. The dense PU layer with high mechanical strength acts as a hydrophobic barrier, which synergistically protects the Li anode from surface corrosion induced by water, oxygen, superoxide anion radicals, and other electrolyte by‐products, while also significantly inhibiting the growth of Li dendrites during long‐term cycling. As a proof of concept, the LOB assembled with this mGP Janus separator exhibits exceptional cycling stability: it can achieve stable cycling for over 800 cycles at a specific capacity of 1000 mAh g^−1^; even at an ultrahigh specific capacity of 3000 mAh g^−1^, the cycle life still exceeds 100 cycles, a performance that ranks among the top tier of state‐of‐the‐art LOBs. At the same time, compared with the control battery using a GF separator, the battery equipped with the mGP separator also demonstrates superior rate capability. The proposed strategy creates an unprecedented route to fabricate high‐performance LOBs systems.

## 2 Results and Discussion

Relevant studies have demonstrated that in the electrolyte of the DMSO/LiClO_4_ system, Li^+^ forms a mononuclear contact ion pair (CIP) with O_2_^−^, wherein the solvation shells tend to overlap, resulting in a significant increase in the overall size of the solvated species and the formation of LiO_2_^−^ solvate complexes with a diameter of approximately 3–5 nm [37, 38, 39, 40]. In contrast, the solvate complex of O_2_^2−^ consists of a binuclear CIP comprising one O_2_^2−^ ion coordinated with two Li^+^ ions. This complex structure requires a larger solvation space to accommodate its configuration, enabling the formation of a more stable three‐dimensional solvation network and thus leading to a larger overall size. Consequently, when the pore size of mSiO_2_ is less than 5 nm, it can effectively inhibit the transport of O_2_^−^/ O_2_^2−^ solvate complexes, whereas pores with larger sizes are ineffective in suppressing the shuttle effect of O_2_^−^/ O_2_^2−^ species [41, 42, 43]. Meanwhile, when the pore size of porous SiO_2_ is less than 1 nm, the transport of solvated Li^+^ is impeded by the strong electrostatic interaction between Li^+^ and the silanol groups on the pore walls, along with the steric hindrance effect within the pore channels, precluding the formation of effective Li^+^ transport pathways [44, 45, 46].

**FIGURE 1:**
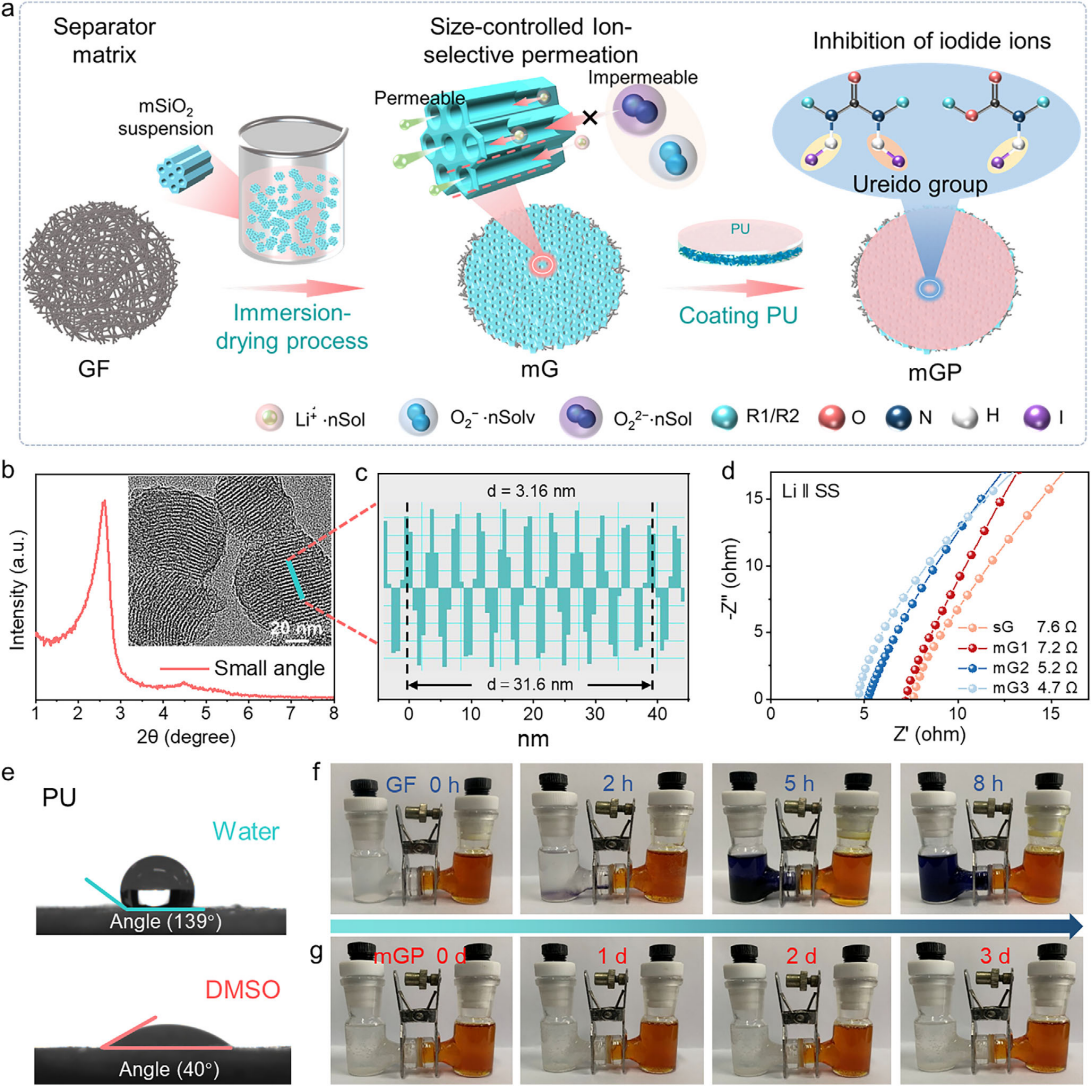
(a) Preparation process of the mGP Janus separator. (b) Small angle XRD of mSiO_2_ as well as high‐resolution TEM image of mSiO_2_ nanoparticles. (c) Average particle size of mSiO_2_. (d) Test of ion transfer resistance for silica/GF separators with different pore sizes. (e) Contact angle test of water and DMSO on the PU side, respectively. Iodide ion blocking test of (f) GF and (g) mGP separators.

In view of this, the pore size of the synthesized mSiO_2_ was precisely tailored to ∼ 3 nm via optimizing the synthesis parameters (Figure S1), aiming to attain superior size‐sieving mediated mass transport performance. Figure 1a illustrates the fabrication process of the mGP Janus separator based on mSiO_2_ nanoparticles. Specifically, the mGP Janus separator is fabricated by immersing a GF separator in an aqueous suspension of mSiO_2_ nanoparticles, followed by a drying procedure. The immersion‐drying process was repeated to ensure full incorporation of mSiO_2_ nanoparticles into the GF matrix, yielding an mSiO_2_/GF (mG) separator. Subsequently, a PU layer was uniformly coated onto one side of the mG separator. The loading levels of mSiO_2_ and PU in the different separators are shown in Figure S2. Notably, the mGP separator can be easily scaled up using the same fabrication protocol (Figure S3). Scanning electron microscope (SEM) image of the in‐house synthesized mSiO_2_ nanoparticles is presented in Figure S4, the mSiO_2_ nanoparticles exhibit spherical and elliptical morphologies, with an average particle size of approximately 191 ± 10 nm (Figure S5). Figure S6 displays the wide‐angle x‐ray diffraction (XRD) pattern of mSiO_2_, revealing a broad characteristic peak around 23°, confirming the amorphous nature of the mSiO_2_ [47]. Figure 1b shows the small‐angle XRD pattern of mSiO_2_ nanoparticles, featuring two diffraction peaks centered at approximately 2.6° and 4.5°, separately corresponding to the (100) and (200) crystal planes. These results verify the presence of an ordered hexagonal mesoporous structure in mSiO_2_ [48]. The inset in Figure 1b shows the transmission electron microscopy (TEM) images of mSiO_2_, Figure 1c shows its pore size distribution curve, with an average pore size of 3.16 nm. The internally aligned parallel pores act as rapid ion transport pathways and enable efficient capture of discharge intermediates throughout LOBs operation.

Figure S7a presents the nitrogen adsorption/desorption isotherm of mSiO_2_, which conforms to a typical type IV isotherm [30]. Figure S7b shows that mSiO_2_ has a narrow pore size distribution. The Brunauer–Emmett–Teller (BET) specific surface area of mSiO_2_ is 1146.3 m^2^ g^−^^1^, with a corresponding pore volume of 0.91 cm^3^ g^−1^. Using the Barrett‐Joyner‐Halenda (BJH) model applied to the adsorption branch, the average pore size of mSiO_2_ is calculated to be 3.16 nm. Figure S8 shows the SEM image of the pristine GF separator, featuring a typically loose structure that impairs its capability to mitigate the shuttle effect of reactive intermediates during the charge–discharge processes of LOBs. Figures S9a–c and S10a display the surface morphology of the mSiO_2_‐functionalized side of the mG separator, where the originally loose and porous voids of the GF separator are fully filled with mSiO_2_ nanoparticles. Figure 1d shows the ionic transport resistance test results of mG separators with different pore sizes, including solid silica/GF (sG), microporous silica/GF (mG1), mesoporous silica (with an average pore size of approximately 3 nm)/GF (mG2), and mesoporous silica (with a pore size > 6 nm)/GF (mG3). The results indicate that sG and mG1 exhibit relatively high ionic transport resistances of 7.6 and 7.2 Ω, respectively. In contrast, mG2 and mG3 present lower and comparable ionic transport resistances of 5.2 and 4.7 Ω, respectively. These findings confirm that mG separators possess excellent ionic transport kinetics when the pore size ranges from 2 to 5 nm, which is consistent with the aforementioned mesopore size screening analyses.

Figures S9d–h and S10b show the morphology of the PU layer, confirming that the opposite side of the mGP separator is uniformly coated with a dense PU resin. This PU layer provides high mechanical strength, as evidenced by a mean modulus of 1.23 GPa (Figure S11), thereby further suppressing the Li dendrite growth. Furthermore, the PU layer can enhance the interfacial contact between the mGP separator and the Li anode, alleviate the volume expansion effect of Li anode, and mitigate the Li corrosion by oxygen reduction reaction (ORR) intermediates [28]. Figure S12 shows the XRD patterns of four separators: GF, mG, GF/PU (GP), and mGP. The amorphous GF exhibits a broad diffraction peak centered at 24.8°, whereas the mG presents a broad shoulder peak at 23.2° corresponding to amorphous silica. Coating with PU shifts this peak to 21.1°. The mGP separator exhibits a broad peak at ∼ 23.3°, which is attributed to the relatively high loading of mSiO_2_. Figure S13 compares the attenuated total reflection Fourier transform infrared (ATR‐FTIR) spectra of the four separators, confirming the co‐existence of mSiO_2_, GF, and PU [49, 50, 51, 52]. As shown in Figure 1e, Figures S14 and S15, the PU layer of the mGP Janus separator exhibits excellent hydrophobicity, with a water contact angle of 139°, while its contact angle with dimethyl sulfoxide (DMSO) is only 40°. For the mSiO_2_‐functionalized side, the contact angles with water and DMSO are 25° and 38°, respectively. These results demonstrate good electrolyte wettability of the mGP separator and can effectively protect the Li anode from water exposure. Figure S16 displays the water permeability of the mGP separator, where the water column height remains constant after 5 h of testing, thus further verifying its excellent water resistance. Figure S17 compares the ionic conductivity (σ) of the mGP separator and other reference separators after immersion in the electrolyte. Although the σ value of the mGP separator is slightly lower than those of GF, mG, and GP counterpart separators, it demonstrates significant superiority over pure PU (less than 2 × 10^−4^ S cm^−1^) and modified Nafion (3.1 × 10^−5^ S cm^−1^) separators [28, 29]. This advantage is attributed to the thin PU layer and the porous structure of mSiO_2_, which facilitate Li^+^ transport and reduce the internal resistance of the separator.

Figure 1f,g and Figure S18 show the capability of different separators to block I_3_^−^ and I^−^ ion diffusion. Notably, after 5 h of testing, I_3_^−^ ions readily permeate through the GF separator, whereas the mGP separator effectively blocks them for over 3 days. A similar trend is observed for I^−^ ions: smooth permeation through the GF separator and obstruction by the mGP separator (Figure S18a,b). Even under a constant current of 0.1 mA over a voltage range of −1.5 to 4.5 V, I^−^ ions barely diffuse through the mGP separator (Figure S18c). The excellent barrier performance of the mGP separator toward iodide species stems from two aspects: on the one hand, it is attributed to the dense physical structure of the PU layer; on the other hand, the ureido groups (─NH─CO─NH─) and carbamate groups (─NH─CO─O─) in the hard segments of PU contain strong hydrogen bond donors (N─H moieties), which can form hydrogen bonds (N─H⋯I^−^) with I^−^ or I_3_^−^ ions. This thus significantly diminishes the shuttling activity of iodine species, enabling the feasible application of iodine‐containing electrolytes [53, 54, 55]. Additionally, Figures S19 and S20 evaluate the electrolyte retention capability of different separators. The GF separator exhibits significant electrolyte evaporation after 36 h, with almost complete evaporation after 60 h (Figure S19a,c). In contrast, the mGP separator maintains good wettability after 36 h and still retains a certain level of electrolyte retention even after 108 h (Figure S19b,d). It is clear that the mGP separator demonstrates significantly enhanced electrolyte retention capability compared to the GF separator. Figure S21 compares the stability of lithium‐nickel foam (Li‐NF) batteries under an oxygen atmosphere, revealing that the mGP separator contributes to mitigating interfacial side reactions. These unique characteristics of the mGP separator are of great significance for its application in LOBs.

**FIGURE 2:**
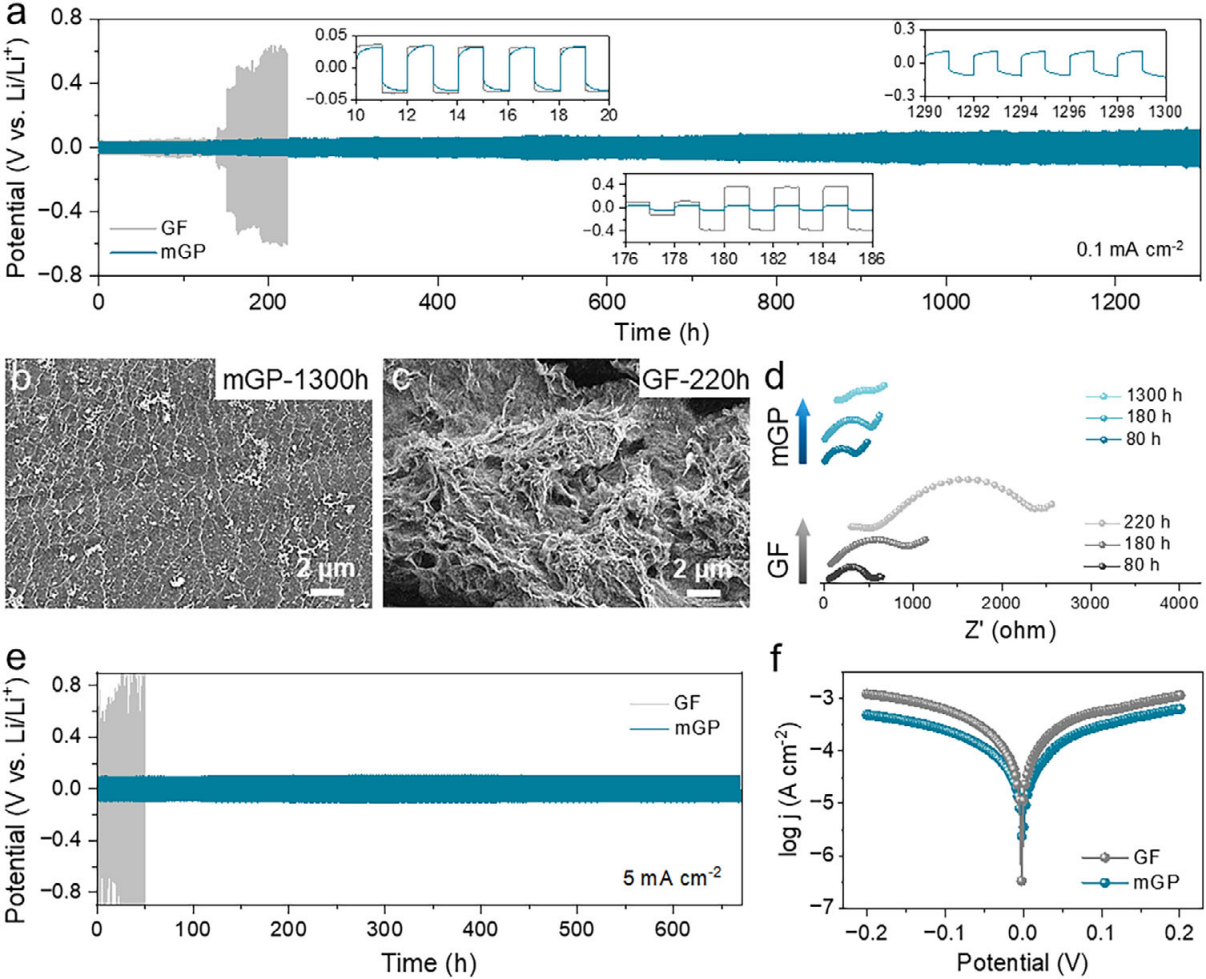
Analyses of Li||Li symmetric cells using GF and mGP separators, respectively. (a) Long‐term cycling performance of the cells. SEM images of the cycled Li anode after different cycles using (b) mGP and (c) GF separators. (d–f) Time‐resolved EIS, high‐rate long‐term cell cycling, and Tafel plots, respectively.

Figure 2a exhibits the long‐term cycling performance (0.1 mA cm^−2^) of Li||Li symmetric cells incorporating GF and mGP separators, respectively. As observed, the former exhibits a sharp potential surge of up to 0.76 V following 180 h of cycling. In contrast, the latter maintains stable cycling behavior for 1300 h with a low polarization potential of merely 0.23 V. Consistently, SEM images in Figure S22 and Figure 2b,c depict the surface morphologies of cycled Li anodes after different cycling durations. The Li anode retains a smooth surface morphology when the mGP‐incorporated Li||Li symmetric cell is tested for 80 h. Even after 1300 h, the Li anode still maintains a relatively smooth surface with no obvious protrusions or dendrites formed (Figure 2b).

In‐depth analyses suggest that the porous structure of mSiO_2_ within the mGP separator, along with the tortuous interparticle pathways, plays a pivotal role in ensuring uniform Li^+^ flux. This enables homogeneous Li^+^ transport and plating/stripping, facilitating stable cell operation. Additionally, the effective combination of mSiO_2_ rigidity and PU's high mechanical strength not only suppresses dendrite growth but also alleviates the volume deformation of the Li anode. On the contrary, apparent protrusions are observed on the surface of the cycled Li anode after 80 h with the GF separator (Figure S22c). Protrusions become more pronounced after 180 h (Figure S22d), accompanied by obvious cracks, which are attributed to non‐uniform Li plating/stripping behavior. By 220 h of operation, distinct dendrites have formed (Figure 2c). All these results further validate the remarkable advantages of the mGP separator over the routine GF counterpart.

To elucidate the underlying mechanisms of the aforementioned phenomena, a series of electrochemical tests were conducted. First, electrochemical impedance spectroscopy (EIS) measurements were performed on cycled Li symmetric cells. As shown in Figure 2d, the impedance values of the mGP‐incorporated Li||Li symmetric cells were approximately 370, 550, and 640 Ω after cycling for 80, 180, and 1300 h, respectively. In contrast, for the GF‑based cells, the impedance reached 540 Ω at 80 h, 930 Ω at 180 h, and sharply increased to 2,300 Ω after 220 h when cell failure occurred. These values are markedly higher than those of the mGP‑incorporated cells at comparable durations. This indicates that the mGP separator exhibits superior interfacial kinetics and the ability to facilitate uniform Li^+^ flux, thereby suppressing Li dendrite growth, which plays a crucial role in enhancing the long‐term cycling stability and rate capability of the cells. Furthermore, as illustrated in Figure 2e, the mGP‐incorporated Li||Li symmetric cell maintained stable cycling for over 600 h even at a high current density of 5 mA·cm^−2^. In contrast, the GF‐based cells failed after merely ∼ 50 h of cycling. Table S1 summarizes the comparison of cycling performance of Li||Li symmetric cells with data reported in other relevant studies. Moreover, Tafel plots (Figure 2f) present that the corrosion current density of the mGP‐incorporated cells is significantly lower than that of the GF‐based cells, confirming superior interfacial compatibility between the Li anode and the mGP separator. These findings further validate that mGP separator effectively facilitates uniform Li deposition/stripping, while it exhibits excellent interfacial compatibility with Li as well as outstanding anti‐polarization capability.

**FIGURE 3:**
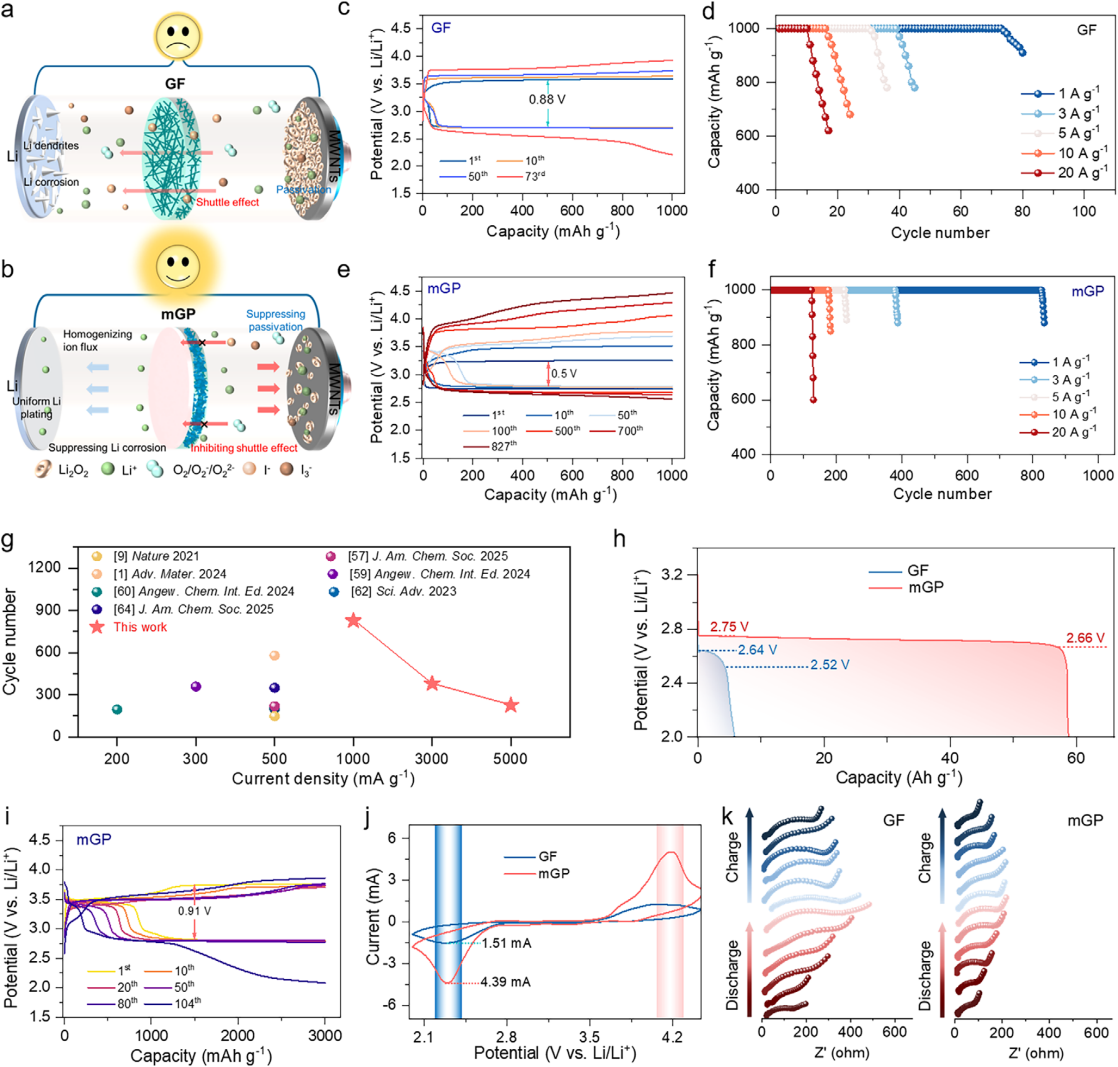
Electrochemical properties of LOBs using GF and mGP separators. Schematic illustration of (a) GF‐based and (b) mGP‐based LOBs. (c, e) Charging‐discharging profiles at different cycles with a constant specific capacity of 1 Ah g^−1^ at 1 A g^−1^ and (d, f) rate performance of (c, d) GF‐based and (e, f) mGP‐based LOBs. (g) Performance comparison of LOBs. (h) Full discharging curves. (i) Cycling curves with a fixed specific capacity of 3000 mAh g^−1^ for the mGP‐based LOB operated at 1 A g^−1^. (j) CV curves and (k) Time‐resolved EIS spectra of LOBs following 30 cycles.

To demonstrate the superiority of the proposed design strategy toward high‐performance separators for LOBs, routine GF and multifunctional mGF separators were utilized to assemble LOBs (Figure 3a,b). As presented in Figure 3c,d and Figure S23a, the routine GF‐based LOB only delivers cycling performance of 73 cycles at a current density of 1 A g^−1^ (2.0–4.5 V) and a fixed specific capacity of 1000 mAh g^−1^. The potential polarization in the first cycle is 0.88 V, and after 73 cycles, the discharge voltage gradually drops to 2.0 V, failing to maintain the rated capacity. In contrast, under the same operating conditions, the mGP‐incorporated LOB achieves an excellent cycling performance of over 800 cycles (Figure 3e,f and Figure S23a), an unprecedented performance for such systems. Even at elevated current densities of 3, 5, 10, and 20 A g^−1^, the LOB retains cycling lives of 380, 226, 177, and 125 cycles (Figure 3f and Figure S23b–e), respectively, which present significant superiority over other counterparts (Figure S24). Notably, the mGF separator enables a remarkably lower initial polarization potential of 0.5 V for the LOB, compared to 0.88 V with the GF separator. Figure 3g and Table S2 summarize the performance comparison of LOBs reported in recent literature [1, 5, 56, 57, 58, 59, 60, 61, 62, 63, 64, 65], highlighting that the mGP‐incorporated LOB in this work exhibits substantial advantages in cycling stability, rate capability, and ultrahigh full discharge capacity. Figure S25 displays the variation of terminal voltages during the discharge/charge cycles of LOBs. In contrast to the GF‐based counterpart, the mGP‐incorporated LOB maintains a relatively stable discharge terminal voltage with increasing cycle number. Figure S26 presents the cycling curves of LOBs using mG and GP separators. Collectively, these results validate the significant advantages of the mGP separator in enhancing the cycling performance of LOBs.

Figure 3h and Figure S27 show the full discharge profiles of LOBs using different separators. Evidently, the mGP separator allows for the highest discharge capacity of 58.77 Ah g^−1^ (relative to mass loading of cathode materials), followed by 36.4 Ah g^−1^ for mG, 26.57 Ah g^−1^ for GP, and 5.78 Ah g^−1^ for GF, respectively. Figure S28 presents the full discharge curves of LOBs using GF and mGP separators in an argon atmosphere, which deliver specific capacities of merely 12 and 18 mAh g^−1^, respectively. These observations indicate that the ultrahigh discharge specific capacity originates from the ORR. Figure 3i and Figure S29 exhibit the charge/discharge profiles of LOBs with different separators at 1 A g^−1^ under a fixed capacity of 3000 mAh g^−1^. Compared to the GF separator, the mGP separator affords 8‐fold longer cycling life. These results confirm that the mGP separator significantly enhances the electrochemical performance of LOBs. Figure 3j and Figure S30 show the CV curves of LOBs with different separators under an oxygen atmosphere, where peak currents corresponding to GF‐, GP‐, mG‐, and mGP‐based LOBs are 1.51, 2.54, 4.01, and 4.39 mA, respectively. These results indicate the enhanced electrochemical ORR activity in LOBs incorporating mG and mGP separators, highlighting that the porous mSiO_2_ nanoparticles facilitate the electrochemical reaction kinetics of O_2_ and Li^+^ at the cathode. Figure 3k displays the time‐resolved EIS curves of LOBs after 30 cycles. The results indicate that the GF‐based LOBs exhibit relatively higher charge transfer impedance, whereas the mGP‐incorporated LOBs show lower impedance values. This further demonstrates that the mGP separator plays a crucial role in LOBs by effectively mitigating Li anode corrosion, alleviating cathode passivation, suppressing the shuttle effect, and improving the reversibility of the system.

Figure S31a–c shows the surface morphologies of the cycled Li anodes in LOBs incorporating the mGP separator. Similar to the Li||Li symmetric cell, the Li anode maintains a flat and dense surface morphology after different cycles. The cross‐sectional images (inset images) exhibit compact structures, which correlate with the stable cycling performance of the mGP‐incorporated LOB. This stability is primarily ascribed to the high mechanical strength and compactness of the PU layer. In contrast, when the GF separator is used, the Li anode begins to pulverize, with numerous protrusions formed on its surface after one cycle (Figure S31d). After 73 cycles (Figure S31e), the Li anode is completely consumed, which involves Li corrosion and volume expansion effects during the charge/discharge cycling of the LOB. Such morphological evolution of the cycled Li anode in LOBs is consistent with that observed in Li||Li symmetric cells. The XRD patterns in Figure S31f reveal the formation of LiOH (PDF No. 85‐0736) on the cycled Li anode of the GF‐based LOB after 73 cycles. In contrast, the mGP‐incorporated LOB exhibits weaker characteristic peaks of LiOH after over 800 cycles, indicating a lower content of LiOH formation. These findings confirm that the mGP separator effectively mitigates volume deformation and corrosion of the Li anode. Furthermore, as illustrated in Figure S32, the mGP separator retains a stable surface morphology with minimal sedimentation even after 827 cycles (Figure S32a–f), whereas the GF separator is covered with side products such as LiOH after 73 cycles (Figure S32g,h), which impedes Li^+^ transport through the separator.

XRD patterns of both separators after different cycles (Figure S32i) provide further evidence for the advantage of the mGP separator. Figure S33 presents x‐ray photoelectron spectroscopy (XPS) results of the cycled Li anode in LOBs after 15 cycles of operation. Comparative analyses indicate that LiOH is distinctly formed on the Li anode surface when the GF separator is employed, as evidenced by the appearance of its characteristic peaks at 54.5 eV in the Li 1s XPS spectrum (Figure S33b), which indicates the occurrence of Li corrosion. In contrast, the mGP‐incorporated sample exhibits a much weaker XPS characteristic peak for LiOH (Figure S33e), demonstrating effective inhibition of the corrosion effect. These results are further authenticated by the corresponding O 1s XPS spectra, where the GF‐based Li anode displays a significantly higher intensity of the O 1s characteristic peak than the mGP‐based counterpart (Figure S33c,f). Consistent with the aforementioned XRD results, these XPS analyses confirm that the mGP separator effectively suppresses the shuttle effect of dissolved intermediate products and Li anode surface corrosion during LOB operation. The favorable performance of the mGP separator is likely attributed to the unique mesoporous structure of mSiO_2_, which enhances the Li^+^ transport kinetics and mitigates the shuttle effect of a certain amount of dissolved oxygen intermediates. Meanwhile, the PU layer further protects the Li anode from corrosion by oxygen reduction intermediates, thereby significantly improving the overall performance of LOBs.

**FIGURE 4:**
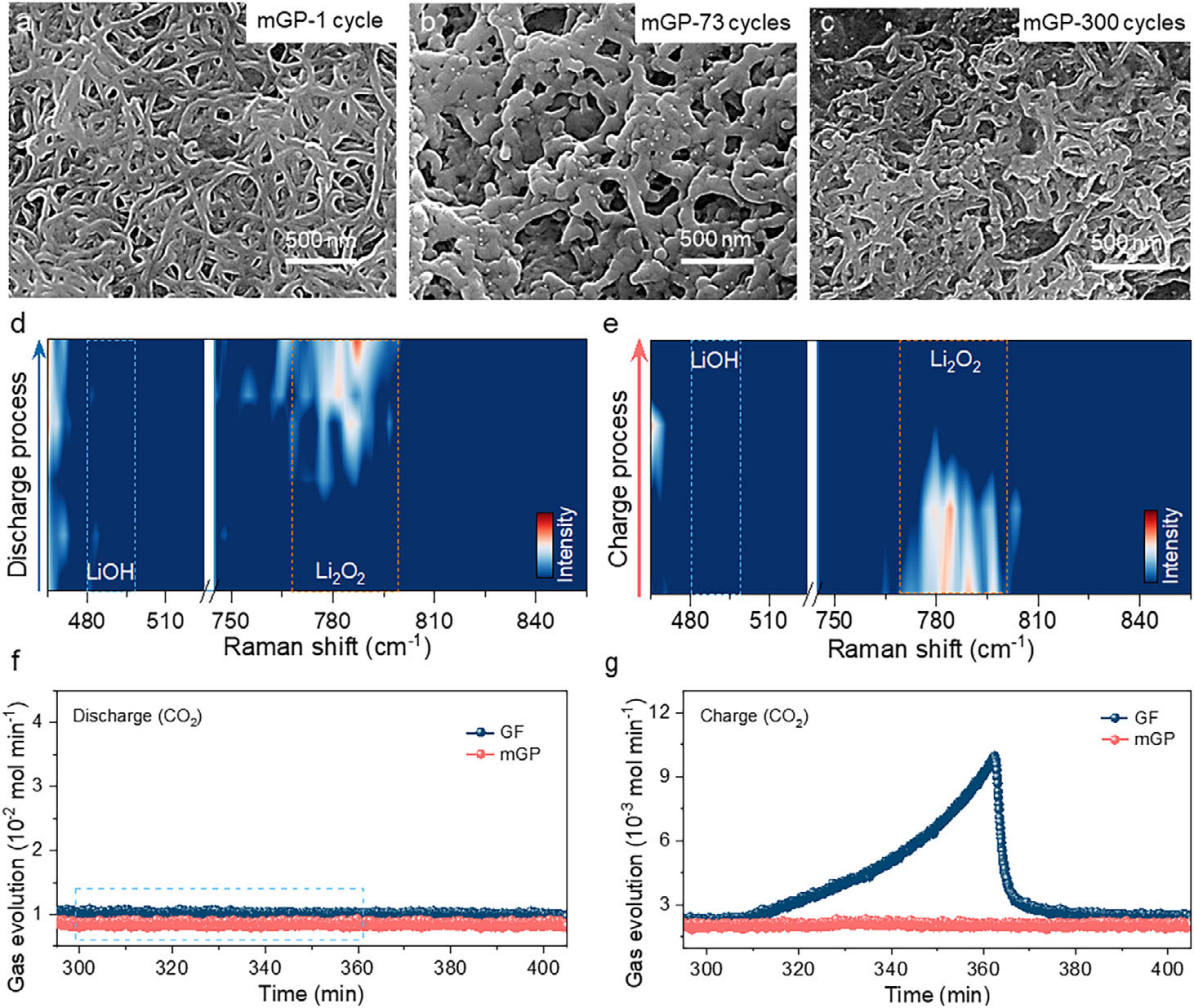
SEM images of the MWCNT cathode in LOBs after different cycles: (a–c) mGP‐based MWCNT cathodes. In situ Raman spectra of the mGP‐based MWCNT cathode under (d) discharging and (e) charging process of LOBs. (f, g) In situ DEMS characterizations of LOBs equipped with various separators.

The SEM image in Figure S34a exhibits the pristine morphology of the multi‐walled carbon nanotube (MWCNT) cathode, which features a well‐defined porous structure composed of strip‐like MWCNTs. When the GF separator is employed in the LOB, as shown in Figure S34b,c, the porous MWCNT cathode is partially covered by irreversible products after one cycle (Figure S34b) and fully blocked by a substantial amount of irreversible products after 73 cycles (Figure S34c), indicating cathode passivation, contributing to the rapid failure of the LOB. Differing from the GF separator, the mGP separator alleviates the formation of these irreversible products and preserves the favorable porous structure of the MWCNT cathode after different cycles (Figure 4a‐c). These observations are further verified by the XRD patterns of the MWCNT cathodes. As shown in Figures S35 and S36, after 73 cycles, the cathode of the GF‐based LOB presents distinct characteristic peaks corresponding to irreversible LiOH products. In sharp contrast, no corresponding characteristic peaks are detected in the mGP‐based cathode within the first 300 cycles (Figure S35), indicating that the mGP separator facilitates the decomposition of products such as Li_2_O_2_.

Figure 4d shows a 2D contour map of in situ Raman spectra of the MWCNT cathode during the discharging process of the mGP‐incorporated LOB. Distinct Li_2_O_2_ peaks centred at 785 cm^−1^ emerge during the discharging process, and their intensity gradually increases. Notably, no characteristic peak corresponding to LiOH is observed during the discharging process, indicating the absence of side reactions. As illustrated in Figure 4e, the Li_2_O_2_ characteristic peaks gradually weaken during the subsequent charge process and fully disappear at the charging terminal, implying the reversible and complete decomposition of Li_2_O_2_ with negligible formation of LiOH byproduct during discharge/charge cycling. For the GF‐based LOB, the MWCNT cathode presents a similar Li_2_O_2_ peak evolution in the in situ Raman spectra (Figure S37). However, additional characteristic peaks centred at 485 cm^−1^, corresponding to LiOH [66, 67], emerge during the discharging process and persist through the subsequent charging process. These phenomena confirm the formation of irreversible LiOH byproduct on the MWCNT cathode of the GF‐based LOB during the discharging process. In situ gas analysis was further performed on LOBs with GF and mGP separators under identical conditions via in situ differential electrochemical mass spectrometry (DEMS), as presented in Figure 4f,g. Neither battery exhibited obvious gas (CO_2_) evolution during the discharge process (Figure 4f, indicating no substantial decomposition of carbon materials at this stage. In contrast, Figure 4g reveals that the GF‐based LOB shows a distinct CO_2_ evolution signal during charging, which demonstrates the decomposition of carbon materials. This phenomenon is attributed to the uneven deposition of discharge products on the cathode, leading to high local current density and subsequent decomposition of the carbon cathode. By comparison, the mGP‐incorporated LOB displayed almost no gas evolution signal under the same conditions. These findings suggest that the mGP separator plays a pivotal role in suppressing side reactions, thereby enabling ultralong cycling stability of LOBs.

**FIGURE 5:**
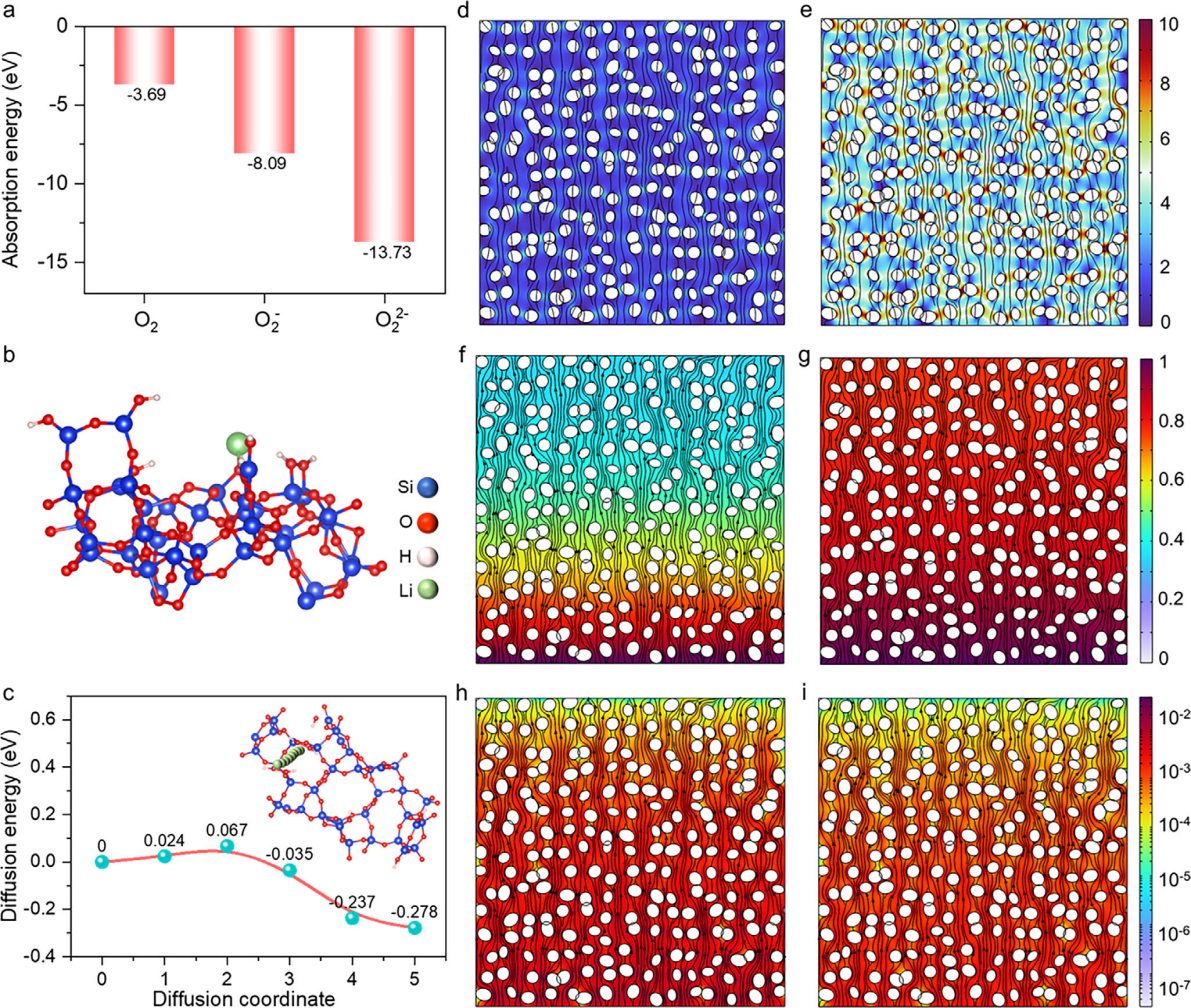
DFT calculations of adsorption energy and diffusion energy on the SiO_2_ surface. (a) Corresponding adsorption energy of O_2_, O_2_^−^, and O_2_^2−^ on the (001) crystal plane of SiO_2_. (b) Interaction site between Li^+^ and SiO_2_, and (c) corresponding diffusion barrier along the optimal path on the (001) crystal plane of SiO_2_. (d–i) Finite element simulation of the physical field on the separator cross‐section: (d, e) the distributions of current density magnitude (mA·cm^−2^), (f, g) ionic molar concentration c (M), and (h, i) total ionic flux magnitude (mol·(m^2^·s)^−1^) for (d, f, h) GF and (e, g, i) mGP, respectively, the scales for both are provided on the right side.

To further corroborate the advantages of the mGP separator, density functional theory (DFT) calculations were employed to elucidate the interactions between mSiO_2_ and reactive species. Figure S38 presents the adsorption structure models of various oxygen species on the (001) crystal plane of SiO_2_. As shown in Figure 5a, the adsorption energies of O_2_, O_2_^−^, and O_2_^2−^ on this plane are calculated to be ‐3.69 eV, ‐8.09 eV, and ‐13.73 eV, respectively. This indicates that the binding interactions between O_2_^−^/ O_2_^2−^ and SiO_2_ are considerably stronger than those between O_2_ and SiO_2_. Consequently, when O_2_^−^ and O_2_^2−^ diffuse in the vicinity of mSiO_2_, they are preferentially captured by the SiO_2_ surface, thereby inhibiting their shuttle between the cathode and Li anode, suppressing interfacial side reactions at the cathode, and alleviating the Li anode corrosion [68]. Figure 5b shows the adsorption sites of Li^+^ on the SiO_2_ (001) plane, with a calculated adsorption energy of −0.91 eV. This indicates an interaction between the Li^+^ solvation shell and the SiO_2_ surface, which modulates the Li^+^ solvation environment to a certain extent. Therefore, free Li^+^ in the electrolyte tends to be adsorbed on the SiO_2_ surface. Notably, the relatively low Li^+^ adsorption energy on SiO_2_ surface is also favorable for fast Li^+^ transport [69, 70, 71, 72, 73]. The Li^+^ diffusion behavior on the SiO_2_ surface was calculated, revealing a relatively low diffusion barrier of 0.067 eV (Figure 5c). This is beneficial to improve Li^+^ diffusion kinetics and ionic flux uniformity, thus boosting the overall electrochemical performance of LOBs [74, 75, 76, 77, 78, 79, 80, 81, 82].

Furthermore, to clarify the ion transport behavior within the separators and the mechanisms underlying the performance enhancement of the mGP separator, finite element simulations were conducted to investigate the real‐time ion distribution dynamics in the cross‐sections of the two separators under an external electric field. As shown in Figure 5d, the GF separator exhibits substantially lower local current density than the mGP separator (Figure 5e). This indicates that the mGP separator enables superior charge transfer efficiency and fast electrochemical reaction kinetics. Figure 5f,g shows the spatial ion concentration distributions in the GF and mGP separators, respectively. The results reveal that the GF separator exhibits a steeper concentration gradient, suggesting a limited ion penetration depth. In contrast, the mGP separator displays a gentler concentration gradient, leading to enhanced ion penetration depth. This confirms fast ion transport within the mGP separator, thereby in turn enabling higher rate capability.

In addition, Figure 5h,i presents a direct comparison of ion flux between the GF and mGP separators. The results suggest that the local ion flux in the GF separator is slightly higher than that in the mGP separator. This indicates that the local ion transport flux in the mGP separator is lower, which in turn indicates more uniform ion flux within the mGP separator. This uniformity is attributed to the well‐defined, uniform nanochannels within the mSiO_2_ component of the mGP separator, which serve to guide ion transport. Consequently, during LOBs cycling, the mGP separator facilitates the formation of more uniform, smaller‐sized discharge products on the cathode side. This markedly enhances the reversibility of LOBs and substantially mitigates side reactions. On the anode side, Li^+^ migrates through the nanochannels and deposits uniformly on the lithium anode under the confinement and directional guidance afforded by the pore channels. This restrains dendrite propagation and substantially homogenizes the kinetics of Li plating and stripping.

## 3 Conclusion

In summary, this work reports a novel multifunctional mesoporous Janus separator with an asymmetric structure, comprising mSiO_2_ nanoparticles with a specific pore size of ∼ 3.16 nm embedded in a GF matrix and a dense PU layer uniformly coated on one side of the GF substrate. The intrinsic nanochannels and high specific surface area of mSiO_2_ enable a size‐sieving mass transport effect and effectively suppress the shuttle effects of reactive intermediates such as O_2_^−^ and O_2_^2−^. Furthermore, the nanochannels inside the mSiO_2_ facilitate the uniform distribution of ionic flux, thereby enhancing the electrochemical reversibility of LOBs. On the other hand, the dense PU layer, featuring excellent hydrophobicity and high mechanical strength, acts as a physical barrier, which not only blocks the Li anode corrosion by water, O_2_, O_2_^−^, and other by‐products but also effectively inhibits Li dendrite growth. Proof‐of‐concept LOBs were assembled with the mGP separator. Electrochemical tests demonstrate that the LOBs achieve an unprecedented cycle life exceeding 800 cycles for the first time at a high specific capacity of 1000 mAh g^−1^, and even at an ultrahigh specific capacity of 3000 mAh g^−1^, the cycle life still exceeds 100 cycles, ranking among the top tier of state‐of‐the‐art LOBs. This work provides an attractive route toward high‐performance semi‐open Li metal battery systems.

## Author Contributions

**Hailiang Mu**: writing – original draft, data curation, formal analysis. **Anlong Liu**: writing – original draft, writing – review and editing. **Kun Luo**: writing – review and editing. **Yaming Pang**: formal analysis. **Chengde Xie**: formal analysis. **Xiaoteng Liu**: writing – review and editing. **Zhihong Luo**: formal analysis. **Guangbin Zhu**: formal analysis. **Yucheng Wang**: funding acquisition. **Qirong Liu**: writing – review and editing, funding acquisition. **Yongbing Tang**: writing – review and editing, funding acquisition, conceptualization, supervision.

## Conflicts of Interest

The authors declare no conflicts of interest.

## Supporting information




**Supporting File**: advs77588‐sup‐0001‐SuppMat.docx.

## Data Availability

The data that support the findings of this study are available from the corresponding author upon reasonable request.
