# Supplementary material for: A Multifunctional Mesoporous Janus Separator With Optimized Mass Transport Regulation Enabling 800‐Cycle Lithium‐Oxygen Batteries
Source: Adv Sci (Weinh). 2026 Sep 13:e77588. Online ahead of print. doi: 10.1002/advs.77588 (PMC13572830; doi:10.1002/advs.77588)
Supplement: Supplementary file 1 [file ADVS-9999-e77588-s001.docx]

**Supporting Information**

**A Multifunctional Mesoporous Janus Separator with Optimized Mass Transport Regulation Enabling 800-Cycle Lithium-Oxygen Batteries**

Hailiang Mu^1,2^, Anlong Liu^3,4^, Kun Luo^1,*^, Yaming Pang^1^, Chengde Xie^2^, Xiaoteng Liu^5^, Zhihong Luo^6^, Guangbin Zhu^7^, Yucheng Wang^1,5,*^, Qirong Liu^2,*^, Yongbing Tang^2,*^

1 Jiangsu Province Engineering Research Centre of Intelligent Manufacturing Technology for the New Energy Vehicle Power Battery, School of Materials Science ＆Engineering, Changzhou University, Changzhou 213164, China. E-mail: luokun@cczu.edu.cn; [longwyc@gmail.com](mailto:longwyc@gmail.com) .

2 Advanced Energy Storage Technology Research Center, Shenzhen Institutes of Advanced Technology, Chinese Academy of Sciences, Shenzhen 518055, China. E-mail: [qr.liu@siat.ac.cn;](mailto:qr.liu@siat.ac.cn;) [tangyb@siat.ac.cn](mailto:tangyb@siat.ac.cn).

3 School of Mechanical Engineering, Shanghai Jiao Tong University, Shanghai, 200240, China.

4 Geely Automobile Research Institute (Ningbo) Co., Ltd., Ningbo, 315336, China.

5 School of Engineering, Physics and Mathematics Faculty of Science and Environment, Northumbria University, Newcastle upon Tyne U.K. NE1 8ST. E-mail: terence.liu@northumbria.ac.uk .

6 Guangxi Key Laboratory of Optical and Electronic Materials and Devices, Guilin University of Technology, Guilin 541004, PR China. E-mail: luozhihong615@glut.edu.cn .

7 School of Environmental Science and Engineering, Tianjin University, Tianjin, 300350, China. E-mail: zhuguangbin@tju.edu.cn .

**1 Experimental section
1.1 Experimental materials and reagents**

Lithium perchlorate (LiClO_4_, 99.99 %), dimethyl sulfoxide (DMSO, 99.9 %), multi-walled carbon nanotubes (MWCNTs, ≥98%), propylene carbonate (PC, 99.7 %), dimethoxyethane (DME, 99.5 %), and lithium iodide (LiI, 99.9 %) were purchased from Sigma-Aldrich. Carbon paper (TGP-H-060) was sourced from TORAY. A borosilicate glass fiber membrane (GF, d = 18 mm) was obtained from Whatman. A metal lithium (Li) disc (d = 14 mm) was purchased from Tianjin Zhongneng Lithium Co., Ltd. Cetyltrimethylammonium bromide (CTAB, analytical reagent, AR), absolute ethanol (analytical reagent, AR), tetraethyl orthosilicate (TEOS, analytical reagent, AR), sodium hydroxide (analytical reagent, AR), N-methylpyrrolidone (NMP, analytical reagent, AR), soluble starch (analytical reagent, AR), and 30 % hydrogen peroxide (analytical reagent, AR) were procured from Sinopharm Chemical Reagent Co., Ltd. Polyurethane (PU, 2805) was supplied by Bayer. The button battery case (CR2032) was obtained from Taizhou Yajun Battery Material Co., Ltd.

**1.2 Battery Assembly and Testing**

Firstly, 2.5 mg multi-walled carbon nanotubes (MWCNTs) are dispersed in 10 mL of absolute ethanol and ultrasonicated for 3 h. Subsequently, the dispersed solution is uniformly sprayed onto a 5×5 cm carbon paper with a loading mass of 0.10 mg cm^-2^. The carbon paper is then dried in a vacuum oven at 85 °C for 20 h and subsequently cut into 1×1 cm square pieces, which serve as the cathode sheets for lithium-oxygen batteries (LOBs). Button cells (CR2032 type, with a cathode shell having 19 holes) are assembled in a glove box (manufactured by Nanjing Jiumen Automation Technology Co., Ltd., where the concentrations of H_2_O and O_2_ are both less than 0.1 ppm) filled with high-purity argon (≈ 99.999 %).

The LOBs tests are conducted in a pure oxygen atmosphere (≥ 99.9%) at an ambient temperature of approximately 25 °C. For the long-cycle test, the current density is set at 1,000 mA g^-1^, and the specific capacity is set at 1,000 mAh g^-1^ (based on the loading mass of MWCNTs). The cut-off voltages are set at 2 V and 4.5 V. For the full-discharge test, the current is 0.1 mA, and the cut-off voltage is set at 2.0 V. The full-discharge capacity is calculated according to the following formula: Full-discharge capacity= (total discharge time × current) / (cathode area × loading amount of MWCNTs). Li||Li batteries were used to investigate the effect of the mSiO_2_ protective layer on the stability of the Li anode. The applied voltage range was from -1.5 V to 1.5 V, the current density was 0.1 mA cm^-2^, and the discharge/charge time was 1 h.

In addition, the ionic conductivities (GF, mGP) were tested using stainless-steel symmetric cells, and the electrochemical impedance spectroscopy (EIS) data were recorded using an electrochemical workstation. The ionic conductivity (σ) was calculated according to the following formula:

$$\sigma=\frac{d}{R_{b}S} (1)$$

Where d represents the thickness of the separator, R_b_ represents the measured resistance, and S = πr^2^ represents the geometric area of the stainless-steel gasket.

Li discs and nickel foam discs (d = 12 mm) were also assembled into button cells (Li-NF) following the same procedures as those for LOBs. Alternating current impedance analysis was carried out in these Li-NF cells to study the stability of Li-NF cells with different separators in oxygen.

**1.3** **Characterization methods**

Morphological and structural analyses were carried out by scanning electron microscopy (SEM, Phenom LE, Thermo Fisher Scientific) and transmission electron microscopy (HRTEM, JEM - 2100, JEOL). X - ray photoelectron spectroscopy (XPS, KRATOS, Axis Ultra^DLD^, UK), X - ray diffractometer (XRD, D/max 2500PC, Rigaku), Fourier transform infrared spectrometer (FTIR, Thermo Scientific Nicolet iS5), nitrogen adsorption/desorption isotherms were recorded by a specific surface area and porosity analyzer (ASAP 2460, Micromeritics), and Raman spectrometer (532 nm, DXR2, Thermo Scientific) were used. Electrochemical impedance analysis was performed using an electrochemical workstation (CHI760E, Shanghai Chenhua Instrument Co., Ltd.). The AC amplitude was 5 mV, and the frequency varied in the range of 0.1 Hz - 10^6^ Hz. Mechanical modulus testing was performed using an Atomic Force Microscope (AFM) (QNM-Young's modulus mapping, Bruker Dimension Icon, Germany).

**1.4** **Preparation Process of mGP Janus Membrane**

A GF separator is immersed in an aqueous suspension of mSiO₂ nanoparticles for 1 min, followed by drying at 85 °C for 3 h. The immersion-drying cycle is repeated 4 times to ensure complete incorporation of mSiO₂ nanoparticles into the GF separator. An mSiO_2_/GF (mG) separator is then obtained after vacuum-drying at 110 °C for 20 h. To prepare the mGP separator, a PU layer is uniformly coated on one side of the mG separator. After PU coating, the sample is dried at 80 °C for 3 h, and this procedure is repeated 4 times.

**1.5 Details of DFT Theoretical Calculations and Finite Element Physical Field Simulations**

In the density functional theory calculations of this paper, the Vienna Ab initio Simulation Package (VASP) was used. It includes the Projector - Augmented - Wave (PAW) potential to describe the electron - ion interaction. The exchange - correlation function was described by the Perdew Burke Ernzerhof (PBE) generalized gradient approximation (GGA). A plane - wave basis set with a cutoff energy of 450 eV was used. A 3×3×1 k - space grid was applied to cover the total energy. The positions of all atoms were adjusted until the inter - atomic force reached 0.02 eV/Å. The adsorption energy was calculated using the supercell model of the SiO_2_ (001) crystal plane (2×2×1).

In this simulation, COMSOL Multiphysics 6.3 software was used for modeling and simulation. For macro-analysis, the Electric Currents physics field from the AC/DC Module and the Dilute Species Transport physics field from the Chemical Species Transport Module were employed. The advantages of using COMSOL software for simulation analysis in this study lie in its rich and diverse physics interfaces—facilitating the selection of appropriate physics fields—and the intuitive visualization of post-processing result graphs.

Geometry creation is a fundamental step in model building, which involves defining the physical shape and spatial layout of the model. Based on the complexity of the model in this simulation, a 2D spatial dimension was selected. In this simulation, the geometric structure constructed was a cross-section consisting of a rectangle with embedded ellipses: the rectangular region served as the simulation cross-sectional area, while the ellipses represented the cross-sections of truncated fibers. The results under steady-state conditions were obtained through analysis.

In the electric currents physics field, the normal current density was set, and current conservation was maintained within the region. By conducting simulation analysis on the geometry, the electric field distribution on the electrode surface was obtained. The relevant formulas are as follows:

$\begin{matrix} \nabla\cdot\mathbf{J}=Q_{j,v} \\ \mathbf{J}=\sigma\mathbf{E}+\mathbf{J}_{e} \\ \mathbf{E}=-\nabla V \end{matrix}$ (2)

Among them: J represents the current density (A m^-2^); Q_j,v_ represents the charge density (C m^-3^); σ represents the electrical conductivity (S m^-1^); E represents the electric field strength (V m^-1^); V represents the electric potential (V).

In electrochemical systems, changes in electric potential can affect the migration of substances. This necessitates the selection of the Dilute Species Transport physics field from the Chemical Species Transport Module, with consideration of electromigration diffusion—specifically the Nernst-Planck equation, which integrates the effects of Fick's laws of diffusion and ionic migration:

$\nabla\cdot\mathbf{J}=R$ (3)

Among them: J is the diffusion flux (mol/(m^2^·s)); R is the source term (mol/(m^3^·s)), which represents the molar generation or consumption rate per unit volume caused by chemical reactions or sources/sinks.

$\mathbf{J}=-D\nabla c-zuFc\nabla V$ (4)

Among them: J represents the substance flux (mol/(m^2^·s)); D represents the substance diffusion coefficient (m^2^/s); c represents the substance concentration (mol/m^3^); z represents the charge of ionic substances; u represents the mobility of charged substances (m^2^/(V·s)); F represents the Faraday constant (C/mol); V represents the electric potential (V).

The mesh is a key factor in computational accuracy. While ensuring simulation precision, the mesh must avoid unnecessary computational load to improve computational efficiency. In this study, triangular meshes were used to construct the overall mesh (Figure S27), with the following geometric mesh parameters: number of mesh nodes is 42,743, number of mesh elements is 84,697, minimum element quality is 0.08842, average element quality is 0.8048.

Through the research, visualized post-processing graphs and related data of current density, electric field magnitude, concentration, and flux were obtained. The distribution of current density helps understand the flow path and spatial distribution of current in the material; the distribution graph of electric field magnitude facilitates identifying regions with concentrated electric fields, thereby avoiding material damage or energy loss caused by excessively strong electric fields; the concentration graph shows the mass distribution of different substances in the system as they change with time and space; and the mass transfer flux graph displays the distribution of mass flux in the system (i.e., the rate and direction of mass transport), which helps understand how substances are transported in the system through mechanisms such as diffusion and migration.

**2 Supplementary Figures**

**Figure S1.** Schematic preparation process of mSiO_2_ nanoparticles.

**Figure S2.** Statistical mass of different separators.

**Figure S3.** Optical photos of large (a) mGP and (b) GF separators with 12 cm in diameter.

**Figure S4.** SEM morphology image of mSiO_2_ nanoparticles.

**Figure S5.** Size distribution of mSiO_2_ nanoparticles.

**Figure S6.** Wide angle XRD of mSiO_2_ nanoparticles_。_

**Figure S7.** (a) Nitrogen adsorption and desorption isotherm of mSiO_2_ nanoparticles. (b) Pore size distribution curve of mSiO_2_ nanoparticles.

**Figure S8.** SEM image of the pristine GF separator.

**Figure S9.** SEM surface morphologies of various separators: (a) pristine GF separator; (b-c) mG separators obtained by soaking pristine GF with mSiO_2_ nanoparticles for 1 and 2 times, respectively; (d-e) mGP separators fabricated by coating mG separators with PU for 1 and 2 times, respectively; (f-h) GP separators prepared by coating pristine GF with PU for 1, 2 and 4 times, respectively.

**Figure S10.** SEM images of the (a) mSiO_2_ side of the mG separator, (b) PU side of the mGP separator.

**Figure S11.** Mechanical modulus of PU layer probed by AFM.

**Figure S12.** The XRD patterns of four different separators.

**Figure S13.** Attenuated total reflection-Fourier transform infrared (ATR-FTIR) analyses of GF, mG, GP, and mGP separators.

As shown in Figure S13, the commercial GF separator exhibits symmetric Si-O stretching vibration at 782 cm^-1^ and asymmetric Si-O-Si stretching vibration at 986 cm^-1^. After filling with mSiO_2_ nanoparticles (mG), three new peaks appear at 1052 cm^-1^, 1636 cm^-1^, and 3406 cm^-1^, corresponding to the asymmetric stretching of Si-O-Si, the bending vibration of H-O-H, and the O-H stretching of surface silanols, respectively.^[1]^ After painting the GF with PU (GP), a series of new peaks are generated. The signal peaks at 1535 cm^-1^, 1732 cm^-1^, and 3337 cm^-1^ are attributed to the C-H stretching, C=O stretching, and N-H stretching vibrations of the PU group, respectively. The resonances at 2875 cm^-1^ and 2961 cm^-1^ are related to the asymmetric and symmetric stretching vibrations of methyl and methylene groups ^[2]^. mGP shows the main peaks of the three components at 782 cm^-1^, 986 cm^-1^, 1052 cm^-1^, 1535 cm^-1^, 1636 cm^-1^, and 1732 cm^-1^ respectively, indicating the co-existence of mSiO_2_ nanoparticles, GF, and PU.

**Figure S14.** Contact angle test of water and DMSO on the mSiO_2_ sides.

**Figure S15.** (a) Water and (b) DMSO contact angle test of the GP separator.

**Figure S16.** Water permeability of the mGP separator. There is no significant drop in the liquid level was observed after 5 h.

**Figure S17.** Comparison of ionic conductivities of GF, mG, GP, and mGP separators immersed in the electrolyte.

**Figure S18.** I^-^ blocking experiments for GF and mGP separators: (a-b) I^-^ diffusion through (a) GF and (b) mGP separators, respectively. (c) For mGP under an electric field, with a current of 0.1 mA, the voltage cycles between -1.5 V and 4.5 V, and the duration is as shown in the figure.

Figure S18 shows the results of iodide ion diffusion experiments for GF and mGP. The left side of the H-cell is filled with 1 M LiClO_4_/DMSO electrolyte, and the right side is filled with 0.05 M LiI in 1 M LiClO_4_/DMSO electrolyte. The inset shows a 0.5 g 10^-1^ mL aqueous solution reagent of starch/H_2_O_2_. Figure S18a demonstrates the static I^-^ diffusion experiment for GF. Before 2 h, the starch/H₂O₂ solution in the inset is transparent, indicating that there is no obvious permeation of I^-^ ions through the GF separator. After 5 h, the starch/H_2_O_2_ solution in the inset begins to become blue, indicating that a large amount of I^-^ ions successfully permeate through the GF separator and into the DMSO electrolyte after standing for 5 h. After 8 h, the amount of I^-^ ion permeated through the GF separator further increases, resulting in obvious blue precipitation. In contrast, there is no obvious precipitation after performing the permeation of I^-^ diffusion through the mGP separator for 4 days (Figure S18b), indicating that the mGP separator has a significant blocking effect on the diffusion of I^-^ ions. Furthermore, an electric field was applied to drive the diffusion of I^-^ ions in the H-cell using the mGP separator. As shown in Figure S18c, even under the action of an electric field, mGP can still maintain its blocking ability to the I^-^ diffusion for at least 4 days.

**Figure S19.** (a-b) Electrolyte retention tests of the pristine GF and mGP separators, as well as (c-d) their corresponding EIS tests.

**Figure S20.** (a-b) Electrolyte retention tests of mG and GP separators, and their corresponding EIS results (c-d).

**Figure S21.** (a-b) EIS analyses of Li-NF batteries with GF and mGP separators exposed to oxygen atmosphere.

**Figure S22.** Analyses of Li||Li symmetric cells using GF and mGP separators, respectively. SEM images of the cycled Li anode after different cycles using (a-b) mGP and (c-d) GF separators.

**Figure S23.** (a) Long-cycle performance curves of LOBs equipped with GF and mGP separators; (b-e) Charge-discharge profiles at different cycles operated at a constant specific capacity of 1 Ah g^-1^ when the mGP-based LOB operated at different current densities.

**Figure S24.** Charge-discharge profiles at different cycles operated at a constant specific capacity of 1 Ah g^-1^ when the GF-based LOBs operated at different current densities.

**Figure S25.** (a) Discharged and (b) charged terminal voltage variation of LOBs with GF and mGP separators at different cycles.

**Figure S26.** Charge-discharge profiles of LOBs using (a) mG and (b) GP separators at different cycles, respectively.

**Figure S27.** Full discharge curves of LOBs assembled with different separators under oxygen atmosphere.

**Figure S28.** Full discharge diagram of LOBs using GF and mGP separators in Ar atmosphere.

**Figure S29.** Cycling curves with a fixed specific capacity of 3,000 mAh g^-1^ for the GF-based LOB operated at 1 A g^-1^.

**Figure S30.** CV curves of LOBs using GP and mG separators.

**Figure S31.** Surface morphologies and cross-sectional images of the cycled Li anode in the (a-c) mGP-based and (d-e) GF-based LOBs after different cycles. (f) XRD patterns.

**Figure** **S32.** (a-h) SEM images of the separator surfaces after long-term operation process of LOBs using (a-f) mGP and (g-h) GF separators, respectively. (i) Their corresponding XRD patterns closed to the Li anode.

**Figure S33.** XPS analysis of the Li surface after 15 cycles of LOBs operation are as follows: (a-c) show the XPS spectra of C1s, Li1s and O1s for GF-based samples, respectively; (d-f) show the XPS spectra of C1s, Li1s and O1s for mGP-based samples, respectively.

**Figure S34.** SEM images of the MWCNT cathode in LOBs after different cycles: (a) The pristine cathode, (b-c) GF-based MWCNT cathode, respectively.

**Figure S35.** XRD patterns of the pristine and cycled MWCNT cathodes.

**Figure S36.** (a) SEM images and (b) XRD pattern of the cycled MWCNT cathode in the mGP-based LOB after 827 cycles.

**Figure S37.** In-situ Raman spectra of the GF-based MWCNT cathode under (a) discharge and (b) charge processes of LOBs.

**Figure S38.** (a-c) are the adsorption models used for O_2_, O_2_^-^, and O_2_^2-^ on SiO_2_ in the DFT calculations, respectively.

**Figure S39.** The triangular mesh used in finite element simulation calculations.

**Table S1.** Comparison of operating parameters and performance of different Li||Li symmetric batteries.

| Configuration of Li\|\|Li symmetric batteries | Current density  (mA cm^-2^) | Lifespan (hours) | Cut-off capacity (mAh) | Ref. |
| --- | --- | --- | --- | --- |
| Janus separator | 0.1 | ≥ 1300 | 0.1 | This work |
|  | 5 | ≥ 600 | 5 |  |
| Single ion separator | 0.1 | ≥ 700 | 0.1 | [4] |
| Interactions in the electrolyte | 0.1 | 2500 | 0.1 | [8] |
|  | 0.4 | 550 | 0.4 |  |
| Non-Newtonian fluid quasi-solid electrolyte | 1 | 2000 | 1 | [15] |
| Quasi-Homogeneous Catalysts | 0.3 | 300 | 0.3 | [16] |
| Manipulating the diffusion energy barrier | 1 | 1000 | 1 | [17] |

**Table S2.** Comparison of operating parameters and performance of different LOBs.

| Configuration of LOBs | Current density  (mA g^-1^) | Lifespan | Cut-off capacity (mAh g^-1^) | Full discharge capacity (mAh g^-1^) | Ref. |
| --- | --- | --- | --- | --- | --- |
| Janus separator，  Li\|mGP\|MWCNT | 1,000 | 827 | 1,000 | 58,768 | This work |
|  | 3,000 | 380 | 1,000 | / |  |
|  | 5,000 | 226 | 1,000 | / |  |
| Zeolite electrolyte solid-state，  Li\|LiXZM\|CNT | 500 | 149 | 1,000 | / | [3] |
| Single ion separator,  Li\|MSP\|MWCNT | 1,000 | 210 | 1,000 | 17,538 | [4] |
|  | 3,000 | 139 | / | / |  |
|  | 5,000 | 107 | / | / |  |
| Interlaced catalytic surface,  Li\|GF\|ISC | 500 | 580 | 500 | 30,923 | [5] |
| Reduce side reactions,  Li\|GF\|CB | 500 | 220 | 500 | 10,932 | [6] |
| Electrocatalytic performance,  Li\|GF\|CC | / | / | 1,000 | 37,462.5 | [7] |
| Interactions in the electrolyte,  Li\|GF\|CP | 500 | 359 | 1,000 | 14,700 | [8] |
| Deep eutectic electrolyte，  Li\|GF\|CNT | 200 | 196 | 500 | 15,225 | [9] |
| Cathode Catalyst，  Li\|GF\|KB | 500 | 425 | 600 | 13,596.2 | [10] |
|  | 1,000 | 407 | / | / |  |
| Cathode Catalyst，  Li\|GF\|COF | 500 | 200 | 1,000 | 17,104 | [11] |
| Molten-salt electrolytes，  Li\|GF\|COF | 1 mA cm^-1^ | 250 | 5 | ≈ 100 | [12] |
| Cathode Catalyst，  Li\|GF\|TBPA | 500 | 350 | 1,000 | / | [13] |
| Cathode Catalyst，  Li\|GF\| In-O- MoS₂@Ru | 200 | 284 | 1000 | / | [14] |

**References**

[1] V. G. Pol, S. V. Pol, P. P. George, B. Markovsky, A. Gedanken, Synthesis of a Conducting SiO_2_-Carbon Composite from Commercial Silicone Grease and Its Conversion to Paramagnetic SiO_2_ Particles. *Phys. Chem. B* **2006**, 110, 13420-13424.

[2] C. Xu, Y. Huang, L. Tang, Y. Hong, Low-Initial-Modulus Biodegradable Polyurethane Elastomers for Soft Tissue Regeneration. *ACS Appl. Mater. Interfaces* **2017**, 9, 2169-2180.

[3] X. Chi, M. Li, J. Di, P. Bai, L. Song, X. Wang, F. Li, S. Liang, J. Xu, J. Yu, A highly stable and flexible zeolite electrolyte solid-state Li-air battery. *Nature* **2021**, *592*, 551-557.

[4] W. Chen, Z. Luo, X. Zhuge, Z. Ding, Y. Ren, A. Loya, Y. Li, K. Luo, Protecting lithium anode with ionic liquid modified poly(vinylidene fluoride) single ion conducting separators for iodide-assisted lithium oxygen batteries. *J. Energy Storage.* **2022**, *50*, 104580.

[5] Z. Sun, X. Lin, C. Wang, Y. Tan, W. Dou, A. Hu, J Cui, J. Fan, R. Yuan, M. Zheng, Q. Dong, Constructing an Interlaced Catalytic Surface via Fluorine-Doped Bimetallic Oxides for Oxygen Electrode Processes in Li-O_2_ Batteries. *Adv. Mater*. **2024**, *36*, 2404319.

[6] Y. Zhang, C. Chen, C. He, Q. Yang, X. Tan, Z. Xu, Y. Jiang, M. Yuan, C. Nan, C. Chen, Atomically Dispersed Ta-O-Co Sites Capable of Mitigating Side Reaction Occurrence for Stable Lithium-Oxygen Batteries. *J. Am. Chem. Soc*. **2025**, 147, 4578.

[7] M. Huang, L. Song, N. Wang, Y. Fu, R. Ren, Z. Li, Y. Lu, J. Xu, Q. Liu, Harnessing 4f Electron Itinerancy for Integrated Dual-Band Redox Systems Boosts Lithium-Oxygen Batteries Electrocatalysis. *Angew. Chem. Int. Ed.* **2025**, 64, e202414893.

[8] D. Y. Yang, J. Y. Du, Y. Yu, Y. Q. Fan, G. Huang, X. B. Zhang, H. J. Zhang, Stable Lithium Oxygen Batteries Enabled by Solvent-diluent Interaction in N,N-dimethylacetamide-based Electrolytes. *Angew. Chem. Int. Ed.* **2024**, 63, e202403432.

[9] W. Sun, F. Zhang, J. Lai, B. Li, X. Hu, B. Gui, N. Chen, X. Guo, Zhujie Li, N. Chen, L. Li, F. Wu, R. Chen, A Supramolecular Deep Eutectic Electrolyte Enhancing Interfacial Stability and Solution Phase Discharge in Li-O_2_ Batteries. *Angew. Chem. Int. Ed*. **2024**, 63, e202409965.

[10] D. Zhang, G. Zhang, R. Liu, R. Yang, X. Li, X. Zhang, H. Yu, P. Zhang, B. W. Li, H. Hou, Z. Guo, F. Dang, Mutually Activated 2D Ti_0.87_O_2_/MXene Monolayers Through Electronic Compensation Effect as Highly Efficient Cathode Catalysts of Li-O_2_ Batteries. *Adv. Funct. Mater*. **2025**, 35, 2414679.

[11] S. Ke, W. Li, Y. Gu, J. Su, Y. Liu, S. Yuan, J. Zuo, J. Ma, P. He, Covalent organic frameworks with Ni-Bis(dithiolene) and Co-porphyrin units as bifunctional catalysts for Li-O_2_ batteries. *Sci. Adv*. **2023**, 9, eadf2398.

[12] Y. Zhu, G. Leverick, L. Giordano, S. Feng, Y. Zhang, Y. Yu, R. Tatara, J. Lunger, Y. Shao-Horn, Nitrate-mediated four-electron oxygen reduction on metal oxides for lithium-oxygen batteries. *Joule* **2022**, 6, 1887-1903.

[13] Z. Jiang, B. Wen, Y. Huang, Y. Wang, H. Fang, F. Li, Heavy Atom-Induced Spin-Orbit Coupling to Quench Singlet Oxygen in a Li-O_2_ Battery. *J. Am. Chem. Soc*. **2025**, 147, 10992-10998.

[14] P. Wang, D. Zhao, P. Zhang, X. Hui, Z. Zhang, R. Wang, C. Wang, X. Ge, Y. Li, L. Yin, P-block element modulated 1 T phase MoS_2_ with Ru lattice grafting for high-performance Li | |O_2_ batteries. *Nat. Commun*. **2025**, 16, 1453.

[15] G. Zheng, T. Yan, Y. Hong, X. Zhang, J. Wu, Z. Liang, Z. Cui, L. Du, H. Song, A non-Newtonian fluid quasi-solid electrolyte designed for long life and high safety Li-O_2_ batteries. *Nat. Commun.* **2023**, 14, 2268.

[16] H. Zhang, Y. Yu, D. Yang, Y. Fan, H. Chen, N. Zhang, Z. Wang, G. Huang, X. Zhang, Multifunctional Quasi-Homogeneous Catalysts as a New Catalytic Strategy to Boost the Performance of Li-O_2_ Batteries. *Adv. Mater.* **2025**, 37, 2413948.

[17] J. Pokharel, A. Cresce, B. Pant, M. Y. Yang, A. Gurung, W. He, A. Baniya, B. S. Lamsal, Z. Yang, S. Gent, X. Xian, Y. Cao, W. A. Goddard, K. Xu, Y. Zhou, Manipulating the diffusion energy barrier at the lithium metal electrolyte interface for dendrite-free long-life batteries. *Nat. Commun.* **2024**, 15, 3085.
